# c-kit^pos^ GATA-4 High Rat Cardiac Stem Cells Foster Adult Cardiomyocyte Survival through IGF-1 Paracrine Signalling

**DOI:** 10.1371/journal.pone.0014297

**Published:** 2010-12-13

**Authors:** Nanako Kawaguchi, Andrew J. Smith, Cheryl D. Waring, Md Kamrul Hasan, Shinka Miyamoto, Rumiko Matsuoka, Georgina M. Ellison

**Affiliations:** 1 International Research and Educational Institute for Integrated Medical Sciences (IREIIMS), Tokyo Women's Medical University, Tokyo, Japan; 2 Stem Cell and Molecular Physiology Laboratory, Research Institute for Sport and Exercise Sciences, Liverpool John Moores University, Liverpool, United Kingdom; 3 Department of Cardiovascular Surgery, Heart Institute of Japan, Tokyo Women's Medical University, Tokyo, Japan; University of Birmingham, United Kingdom

## Abstract

**Background:**

Resident c-kit positive (c-kit^pos^) cardiac stem cells (CSCs) could be considered the most appropriate cell type for myocardial regeneration therapies. However, much is still unknown regarding their biological properties and potential.

**Methodology/Principal Findings:**

We produced clones of high and low expressing GATA-4 CSCs from long-term bulk-cultured c-kit^pos^ CSCs isolated from adult rat hearts. When c-kit^pos^ GATA-4 high expressing clonal CSCs (cCSCs) were co-cultured with adult rat ventricular cardiomyocytes, we observed increased survival and contractility of the cardiomyocytes, compared to cardiomyocytes cultured alone, co-cultured with fibroblasts or c-kit^pos^ GATA-4 low expressing cCSCs. When analysed by ELISA, the concentration of IGF-1 was significantly increased in the c-kit^pos^ GATA-4 high cCSC/cardiomyocyte co-cultures and there was a significant correlation between IGF-1 concentration and cardiomyocyte survival. We showed the activation of the IGF-1 receptor and its downstream molecular targets in cardiomyocytes co-cultured with c-kit^pos^ GATA-4 high cCSCs but not in cardiomyocytes that were cultured alone, co-cultured with fibroblasts or c-kit^pos^ GATA-4 low cCSCs. Addition of a blocking antibody specific to the IGF-1 receptor inhibited the survival of cardiomyocytes and prevented the activation of its signalling in cardiomyocytes in the c-kit^pos^ GATA-4 high cCSC/cardiomyocyte co-culture system. IGF-1 supplementation or IGF-1 high conditioned medium taken from the co-culture of c-kit^pos^ GATA-4 high cCSCs plus cardiomyocytes did extend the survival and contractility of cardiomyocytes cultured alone and cardiomyocytes co-cultured with c-kit^pos^ GATA-4 low cCSCs.

**Conclusion/Significance:**

c-kit^pos^ GATA-4 high cCSCs exert a paracrine survival effect on cardiomyocytes through induction of the IGF-1R and signalling pathway.

## Introduction

Heart failure remains a leading cause of morbidity and mortality in the Western World [1]. The central cellular mechanism underlying the development of myocardial dysfunction is a decrease in the number of viable cardiomyocytes, secondary to either acute ischemic injury or chronic apoptosis, and an inability of remaining cardiomyocytes to compensate for this loss through a hypertrophic response [2]–[4]. Therefore, it has been a long-term goal to find a method to replace the lost cardiomyocytes by increasing their number and/or to recover cardiac function after myocardial injury. A recent attractive method is the usage of stem cells which upon transplantation into the infarcted myocardium can repair and regenerate the lost tissue and improve cardiac function [5]–[6]. Many types of stem cells have been proposed as suitable candidate cells to repair and regenerate the myocardium after infarction and failure [7]. Despite highly promising initial animal studies [8], the use of bone marrow derived cells (BMDCs) have shown only a modest effect on improving cardiac function following double-blind randomized placebo controlled clinical trials [9]–[10]. These results still bring into question the mechanism of action of BMDCs, with the majority settling for a beneficial remodelling effect through paracrine mechanisms. BMDCs release a complex mixture of cytokines and growth factors involved in cell survival, proliferation and migration [11]–[12]. In essence this means that instead of the cells undergoing cardiomyogenic differentiation, they contribute to improved myocardial contractility through an unidentified paracrine mechanism by the amelioration of ventricular remodelling (decreasing fibrosis, hibernation and stunning), inhibition of the inflammatory response, increasing existing cardiomyocyte survival [13]–[14], increasing angiogenesis and stimulating the activation of resident cardiac stem/progenitor cells to give rise to new vasculature and cardiomyocytes. Together with the controversy that surrounds the cardiomyogenic potential of BMDCs [15]–[17] their potential as being the ‘best’ type and source of cell to reconstitute the myocardium and improve function following damage is questionable.

c-kit positive (c-kit^pos^) cardiac stem cells (CSCs) are the only adult derived cardiac stem/progenitor cells shown to exhibit all the characteristics of *bona fide* stem cells, being clonogenic, self-renewing, multipotent and having substantial regenerative potential in an *in vivo* animal myocardial infarction (MI) regeneration assay [5]. Recently, exogenous cardiac progenitor cell transplantation into the 30 day infarcted rat heart was shown to activate the endogenous cardiac progenitor cells alleviating left ventricular dysfunction [18]. Furthermore, human cardiosphere-derived cells have also been reported to exhibit paracrine effects, through secretion of growth factors and resultant anti-apoptotic effects on surviving cardiomyocytes following their intra-myocardial injection after MI in mice [19]. Therefore, because of their direct regenerative and paracrine effects, the use of CSCs as the most appropriate and optimal candidate cell for future cardiac regenerative medicine strategies are considered highly promising.

We previously reported that c-kit^pos^ CSCs isolated from adult rat hearts and then cultured in the long-term (termed bulk cultured CSCs (CSC-BC)) exhibited significant variability in the expression of stemness and cardiac differentiation potential markers over time [20]. Furthermore, we showed that CSC-BC could extend cardiomyocyte survival in the short-term when grown in a co-culture system and that the growth factors, IGF-1 and VEGF could play a role in this effect [20]. c-kit^pos^ CSCs possess the IGF-1 receptor system [21]-[22] and IGF-1 has a positive effect on cardiomyocyte survival in aging mice [21] and the infarcted pig heart (Ellison et al., unpublished data). Furthermore, IGF-1 release from nanofibers improved in part the recovery of myocardial structure and function after MI in rats [23]–[24].

GATA-4 is a member of the GATA family of zinc finger transcription factors and is an early cardiomyocyte marker, playing an important role in transducing nuclear events that modulate cell lineage differentiation during development [25]–[26] and hypertrophy of adult cardiomyocytes [27]. Recently, we showed that CSC-BC that express high levels of GATA-4 have potent cardiomyocyte differentiation capacity, compared to CSC-BC that express low levels of GATA-4 [20]. Several reports have suggested novel functions of GATA-4, as a regulator of cytokines and growth factors through GATA elements [28]–[29]. Indeed, Heineke et al. (2007) described that GATA-4 acts as a mediator for angiogenesis through enhanced expression level of VEGF, when GATA-4 was over-expressed through adenoviral enhancement [28].

In our continued search for the best type of cell to be used for reparative and regenerative cardiac therapy, here we compared the effects of c-kit^pos^ GATA-4 high clonogenic CSCs (cCSCs) with c-kit^pos^ GATA-4 low cCSCs on adult rat cardiomyocyte survival and contractility in a co-culture system *in vitro*. Next, we determined the signaling pathway involved to explain this pro-survival mechanism of action.

## Materials and Methods

### Animal experiments

Experimental procedures were carried out under the British Home Office Animal (Scientific Procedures) Act 1986 and/or approved by the corresponding Institutional Review Boards. Animal experiments were performed according to Guidelines of Tokyo Women's Medical University on Animal Use, The Principles of Laboratory Animal Care, formulated by the National Society for Medical Research, and Guide for the Care and Use of Laboratory Animals, prepared by the Institute of Laboratory Animal Resources and published by the National Institutes of Health (NIH Publication No. 86-23, revised 1985).

### Isolation of c-kit positive (c-kit^pos^) CSCs and Adult Rat Ventricular Cardiomyocytes

c-kit^pos^ cells were isolated as previously described [5], [20]. Hearts were excised from adult male Lewis or Wistar rats (∼250 g), the aorta cannulated and hung on a retrograde perfusion system [30]. Briefly, this procedure consists of three main steps: 1) A collagenase type II perfusion of the myocardium performed at 37°C with HEPES-MEM, gassed with 85% O_2_ and 15% N_2_. 2) The heart is removed from the apparatus, the atria are removed and discarded, and the ventricles cut into small pieces and the fragments shaken in re-suspension medium at 37°C. 3) Adult ventricular cardiomyocytes and small cardiac cells are separated by centrifugation and then the cardiomyocyte suspension is passed through a BSA size separation gradient for further purification of viable, rod-shaped cardiomyocytes. Cardiomyocytes were used for the co-culture assays. For isolation of c-kit^pos^ CD45^neg^ CSCs, the cardiac small cell fraction was treated with an anti-rat CD45 mouse monoclonal antibody (Biolegend). The CD45 positive cells are depleted from the preparation through indirect anti-mouse IgG microbead sorting (Miltenyi), leaving the CD45^neg^ fraction. The c-kit^pos^ CSCs were enriched from the CD45^neg^ fraction, through incubation with a rabbit anti-c-kit primary antibody (Santa Cruz), followed by goat anti-rabbit antibody conjugated with magnetic microbeads for separation and isolation by AUTOMACS Technology (Miltenyi). The purity of the c-kit^pos^ CSC preparation was verified using FACS and Immuno-cytospin staining [30].

### Cell culture and cloning

c-kit^pos^ CSCs were cultured in complete medium (Comp M) [Dulbecco's Modified Eagles Medium [DMEM] supplemented with 10% embryonic stem cell grade fetal bovine serum (Invitrogen), 5% horse serum (Sigma), 10 ng/mL leukemia inhibitory factor (LIF, Chemicon), 5 U/L Erythropoietin (EPO, Sigma), penicillin-streptomycin (Wako), and fungizone (Wako), and gentamicin (Invitrogen)] at 37°C in 5% CO_2_ incubator and the medium was replaced at 3 to 4 day intervals. c-kit^pos^ CSCs were seeded by serial dilution into single wells of 96 well plates at a density of 0.5 cell per well for the generation of single cell clones [5], [20]. For the generation of GFP positive (GFP^pos^) c-kit^pos^ clonal CSCs (cCSCs), cCSCs were transfected with a lenti-viral vector encoding the green fluorescent protein (GFP), according to the manufacturer's instructions (Invitrogen).

The medium used for co-culture experiments was cardiogenic differentiation medium (CGDM), which was composed of MEM Alpha (GIBCO), 10% FBS, and supplemented with 1 µM dexamethasone (Sigma), 50 µg/ml ascorbic acid (Sigma), and 1 mM β-glycerophosphate (Sigma) [31]. Fibroblast (Fibro) Rat-1 cells were used as a control and were obtained from RIKEN Cell Bank.

### RT-PCR and Quantitative Real Time RT-PCR analysis

RNA was extracted from cardiomyocytes and cloned c-kit^pos^ CSCs using ISOGEN (Wako), Nucleospin (Macherey-Nagel) or Qiagen RNeasy columns. Residual amounts of DNA were removed by on-column DNase treatment using the RNase-Free DNase Set (Qiagen) during the RNeasy procedure. RT-PCR was performed by one step (Invitrogen) or two-step (Applied Biosystems). Cycle conditions were first, 94°C, 15 s, then, 94°C, 30 s for denature, 60°C, 30 s for annealing, and 72°C, 30 s for extension. Cycle number was 30, and 72°C for 7 min until the end. PCR products were run on 4% agarose/1x TBE gel (Reliant gel system, CAMBREX). The expression levels were evaluated by the strength of the signal (the intensity of the band stained with ethidium bromide). Quantitative RT-PCR was performed using SYBR Green (BioRad) on a MyIQ thermocycler (BioRad). The PCR-reaction included 2 µl of template cDNA, and 300 nM forward and reverse primers. PCR efficiency was evaluated by using a standard curve of five serial dilution points. Data were analysed using BioRad IQ software and mRNA was normalized to the housekeeping gene, GAPDH. Primers were designed using the Primer 3 software and the specific sequences are given in Table S1. All reactions were carried out in triplicate.

### Western blot analysis

Immunoblots were carried out using protein lysates obtained from c-kit^pos^ clonogenic CSCs (cCSCs) and co-cultured cardiomyocytes [30]. Generally, aliquots equivalent of ∼50 µg of protein were separated on gradient (6–15%) SDS-polyacrylamide gels. After electrophoresis, proteins were transferred onto nitrocellulose filters, blocked with either 5% dry milk or 5% bovine serum albumin, and incubated with Abs against GATA-4 (Santa Cruz), IGF-1R, phospho-IGF-1R, Akt, phospho-Akt, (Cell Signaling), at dilutions suggested by the manufacturers. Actin (Santa Cruz) was used as a loading control. Proteins were detected by chemiluminescence using horseradish peroxidase-conjugated secondary Abs and the Chemidoc XRS system (Bio-Rad) and optical density (O.D.) was measured.

### cCSC/Cardiomyocyte co-culture and ELISA analysis

c-kit^pos^ GATA-4 high or c-kit^pos^ GATA-4 low cCSCs and adult cardiomyocytes were co-cultured in 6-well plates or 35 mm dishes in CGDM, either with or without culture inserts (Corning); to separate contact between the cCSCs and the cardiomyocytes [20]. The inserts (Nunc) have a pore size of 0.2 µm which does not allow movement of the cCSCs cultured on the insert/membrane into the cardiomyocytes cultured on the substrate (Fig. S1; Reference 20). As control, rat fibroblasts were co-cultured with adult cardiomyocytes. Fibroblasts or cCSCs (1×10^6^ cells/well) were cultured on the inserts and adult cardiomyocytes were cultured on the substrate. Adult cardiomyocytes were cultured at 4×10^4^ cells/well. Adult cardiomyocytes, c-kit^pos^ GATA-4 low clone CSC1A (cCSC1A), c-kit^pos^ GATA-4 low clone CSC3C (cCSC3C), c-kit^pos^ GATA-4 high clone CSC4A (cCSC4A), c-kit^pos^ GATA-4 high clone CSC10A (cCSC10A), or rat fibroblast (Fibro) cells (1×10^6^ cells/well) were also grown alone using the indicated medium. The medium was harvested and changed for fresh CGDM every 3 or 4 days. After 3, 7, 14 and 21 days of co-culture, 3×35 mm dishes/condition were fixed and stained to assess the number of apoptotic myocytes (TdT and caspase-3; at 3 and 7 days) and attached cTnI positive cells (at 7, 14 and 21 days). The number of beating cells was counted in 5 random fields/dish at ×20 magnification and expressed as a percent of total cardiomyocytes. A total of 3 wells were counted/condition. At 3 weeks, the medium was harvested from co-culture conditions with inserts and ELISA assay was performed using kits against specific growth factors, IGF-1, TGF-β1, BMP-2 (R&D Systems), TNFα, (Pierce Biotech. Inc.) and VEGF, (RayBiotech, Inc.), according to the manufacturer's instruction. To inhibit the IGF-1 signaling pathway, cardiomyocytes co-cultured with c-kit^pos^ GATA-4 high cCSC4A using inserts for 21 days were harvested following 48 hours treatment with IGF-1 receptor blocking antibody (1 µg/ml; Abcam) or Akt inhibitor (124005, 10 µmol/l; CalBiochem) added to the culture medium. All assays were performed in triplicate. For experiments assessing the effects of IGF-1, wells/dishes were supplemented with 200 ng/ml of murine IGF-1 (Peprotech).

### Immunofluorescence staining

Cultured cells were fixed with 4% formaldehyde for 20 minutes and then stained. For GATA-4 identification, cells were permeabilized with 0.2% triton-X 100 for 20 minutes, washed with PBS, blocked with 2% BSA (Sigma) for 15 minutes, and then treated with an antibody against GATA-4 (Santa Cruz). Cells were all counterstained with Hoechst (Invitrogen) for nuclei detection. For cell survival, co-cultures were stained for anti-caspase 3 (Abcam) and using the Terminal deoxynucleotidyltransferase (TdT) assay (Invitrogen), at 3 and 7 days. Cardiomyocytes were co-stained for cTnI (Santa Cruz). Nuclei were detected by DAPI (Sigma). The percentage of TdT-positive and caspase-3 positive cardiomyocytes was determined by counting 20 random fields at ×40 magnification for each well, with a total of 3 wells/condition. Numbers were expressed as a percentage of TdT- or caspase-3-positive cardiomyocytes relative to the total number of cardiomyocytes counted. For cell proliferation, BrdU was added, 1 µg/ml every 6 hours to the co-cultures for 7 days. BrdU incorporation was assessed using the BrdU detection kit (Roche) and the cardiomyocytes were co-stained for cTnI (Santa Cruz). Co-cultured cardiomyocytes were also stained for Ki67 (Abcam) and Myosin Heavy Chain (MHC; Sigma) at 7 days. 20 random fields at ×40 magnification were counted for each well, with a total of 3 wells/condition. For c-kit^pos^ cCSC cardiomyogenic differentiation, cultures were stained for GFP (Rockland) and α-sarcomeric actin (Sigma). 20 random fields at ×40 magnification were counted for each dish, with a total of 3 dishes/condition. Numbers were expressed as a percentage of GFP-α-sarcomeric actin postive cells relative to the total number of GFP cells counted. Secondary Dylight antibodies were obtained from Jackson Immunoresearch. Secondary antibody incubation alone was used as a negative control. Immunostaining was visualized and analyzed using epi-fluorescence (Zeiss Axioplan2 and Nikon E1000M) and images acquired with laser scanning confocal microscopy (Zeiss LSM510 META and LSM710).

### Statistical Analysis

Data are reported as Mean ± S.D. Significance was determined by the analysis of variance (ANOVA) or *t* tests. The Bonferroni post hoc method was used to locate the differences. Significance was set at P<0.05.

## Results

### Generation of GATA-4-high and -low expressing single cell derived clonal cultures from bulk cultured c-kit^pos^ Cardiac Stem Cells (CSC-BC)

**Figure 1:**
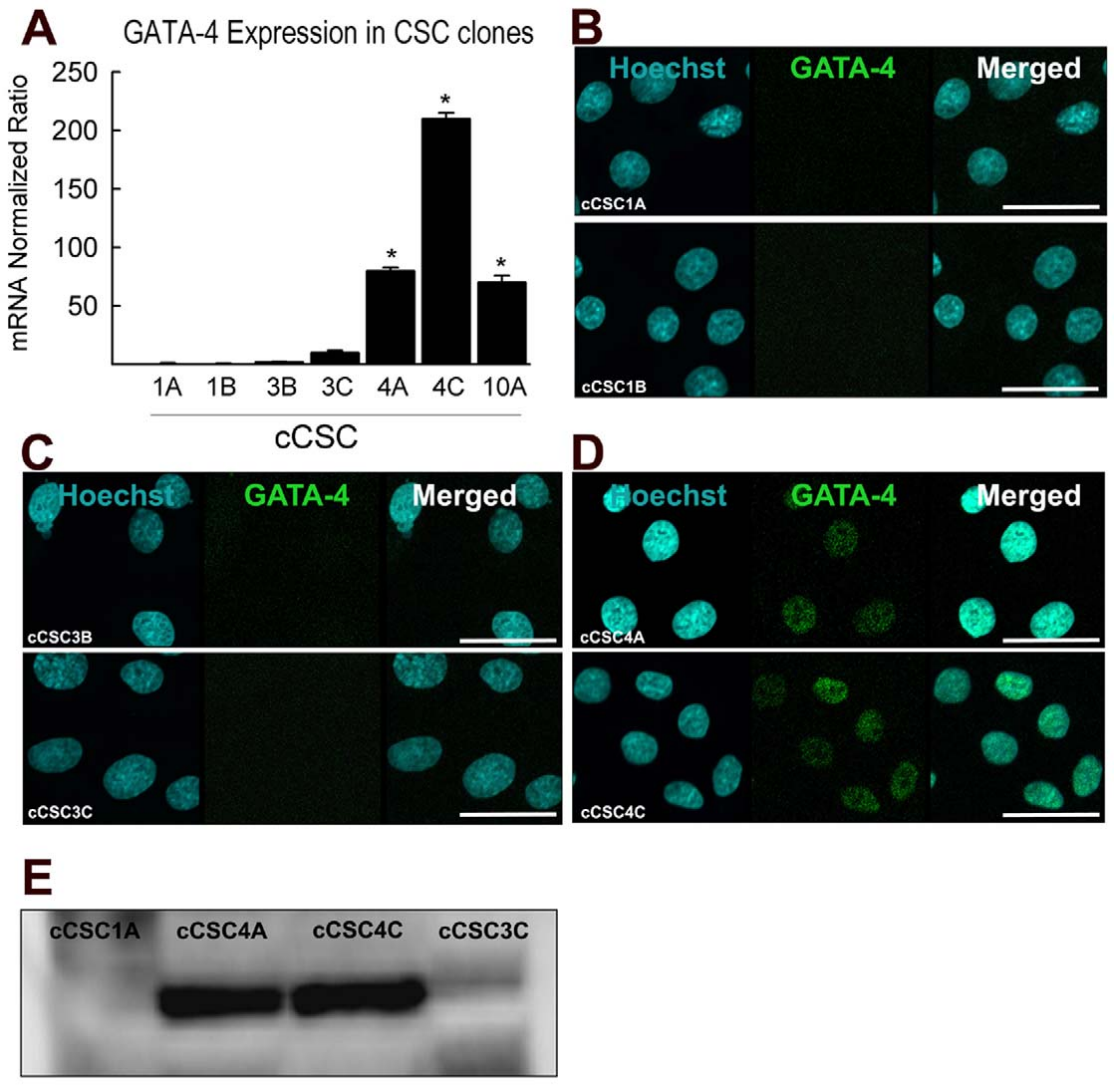
Single Cell Derived GATA4-high and -low c-kit^pos^ CSC Clones. **A**. Quantitative RT-PCR analysis shows that single cell derived clones of c-kit^pos^ CSCs (cCSCs) derived from bulk-cultured CSCs have high (cCSC4A; cCSC4C; cCSC10A) and low expression (CSC-1A, cCSC1B, cCSC-3B, cCSC3C) of GATA-4. *P<0.001 vs. cCSC1A, 1B, 3B and 3C. Data are Mean ± SD of 3 assays (with 3 triplicates/assay) and analysed using ANOVA. **B–D**. Representative immunocytochemical staining, comparing GATA-4 (green) expression of GATA-4 low c-kit^pos^ CSC clones (cCSC1A, cCSC1B, cCSC3B, cCSC3C; B–C) with GATA-4 high c-kit^pos^ CSC clones (cCSC4A, cCSC4C; D). Hoechst identifies Nuclei in blue. Bar = 50 µm. **E**. Western blot analysis confirms increased GATA-4 expression in cCSC4A and cCSC4C compared with low GATA-4 expressing clones, cCSC-1A and cCSC-3C.

We previously described differential expression, at both the mRNA level and protein level, of GATA-4 in long term bulk-cultured c-kit^pos^ CSCs (CSC-BC) [20]. To purify a population of c-kit^pos^ GATA-4 high and c-kit^pos^ GATA-4 low expressing CSCs, we derived single cell clones (cCSCs) from these different CSC-BC and analysed them for GATA-4 expression. The cloned cells from CSC-BC4 (cCSC4A and 4C) and CSC-BC10 (cCSC10A), had enhanced expression levels of GATA-4, which was around 8-200 times higher, compared to clones from CSC-BC1 (cCSC1A and 1B) and 3 (cCSC3B and 3C), when analysed by quantitative RT-PCR (Fig. 1A). We investigated 5 clones out of 40 from CSC-BC4 and all 5 clones had similar high expression levels of GATA-4 (data not shown). The expression level of GATA-4 in the cCSC 1A, 1B, 3B, 3C, 4A and 4C was confirmed by immuno-fluorescence staining (Fig. 1B–D) and Western blot analysis (Fig. 1E). c-kit^pos^ cCSCs were determined as being GATA-4 low if the mRNA ratio normalized to GAPDH was ≤10 with negative or very faint immunostaining or Western blot for GATA-4. c-kit^pos^ GATA-4 high CSCs were defined by an mRNA ratio normalized to GAPDH of ≥70 and positive immunostaining or Western blot for GATA-4. Two c-kit^pos^ GATA-4 low cCSCs populations (cCSC1A and cCSC3C) and two c-kit^pos^ GATA-4 high cCSCs populations (cCSC4A and cCSC10A) were selected for further analyses.

### Cardiac Stem Cells with increased expression of GATA-4 enhance survival and contractility of adult ventricular cardiomyocytes

**Figure 2:**
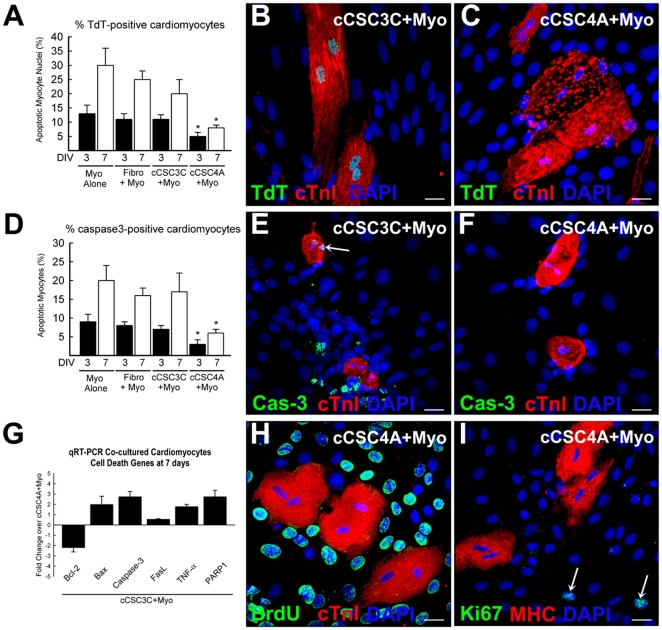
c-kit^pos^ GATA-4 high cCSCs improve survival of adult cardiomyocytes yet do not promote cardiomyocyte proliferation. **A**. The percent number of TdT-positive cardiomyocytes at 3 and 7 days in vitro (DIV) following culture alone (Myo alone) or co-culture with either fibroblasts (Fibro+Myo), GATA-4 low expressing c-kit^pos^ CSC clone (cCSC3C+Myo) or GATA-4 high c-kit^pos^ CSC clone (cCSC4A+Myo). *P<0.05 vs. Myo alone, Fibro+Myo, cCSC3C+Myo. Data are Mean ± SD for 3 wells/condition and analysed using ANOVA. **B–C**. Representative confocal microscopy images showing TdT-positive (green) and TdT-negative cardiomyocytes (red; cTnI) at 7 days following co-culture with c-kit^pos^ cCSC GATA-4 low (cCSC3C+Myo) or c-kit^pos^ cCSC GATA-4 high (cCSC4A+Myo), respectively. Bar = 20 µm. **D**. The percent number of apoptotic, caspase3-positive cardiomyocytes at 3 and 7 days in vitro (DIV) following culture alone (Myo alone) or co-culture with either fibroblasts (Fibro+Myo), GATA-4 low expressing c-kit^pos^ CSC clone (cCSC3C+Myo) or GATA-4 high c-kit^pos^ CSC clone (cCSC4A+Myo). *P<0.05 vs. Myo alone, Fibro+Myo, cCSC3C+Myo. Data are Mean ± SD for 3 wells/condition and analysed using ANOVA. **E–F**. Representative confocal microscopy images showing caspase3-positive (green; arrow) and caspase3-negative cardiomyocytes (red; cTnI) at 7 days following co-culture with c-kit^pos^ cCSC GATA-4 low (cCSC3C+Myo) or c-kit^pos^ cCSC GATA-4 high (cCSC4A+Myo), respectively. Bar + 20 µm. G. Quantitative RT-PCR analysis showing the fold change of Bcl-2, Bax, caspase-3, FasL, TNF-α and PARP1 in cardiomyocytes at 7 days following co-culture with c-kit^pos^ GATA-4 low cCSCs (cCSC3C+Myo), compared to c-kit^pos^ GATA-4 high cCSCs (cCSC4A+Myo). Data are Mean ± SD of 3 assays (with 3 triplicates/assay). **H–I**. Representative confocal microscopy images showing BrdU (H; green) and Ki67 (I; green) negative cardiomyocytes (H; red, cTnI. I; red, MHC) at 7 days following co-culture with c-kit^pos^ GATA-4 high cCSCs (cCSC4A+Myo). Arrows indicate 2 Ki67 (green) positive nuclei in I. Nuclei are stained by DAPI in blue. Bar = 20 µm.

**Figure 3:**
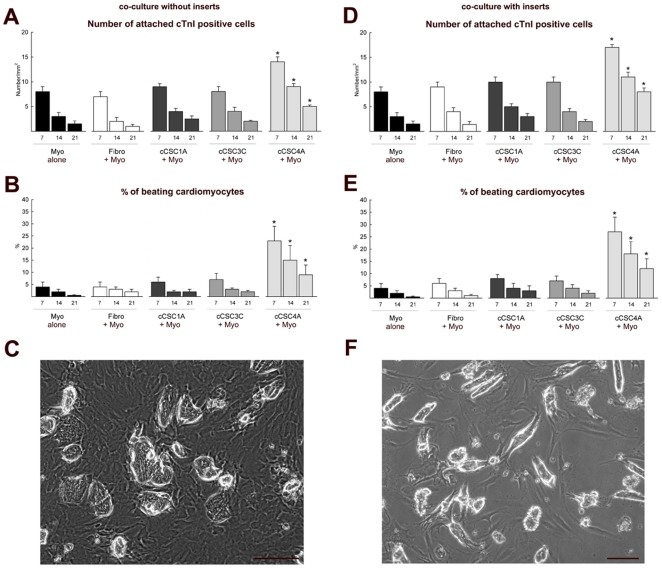
Co-culture of GATA-4 high expressing c-kit^pos^ cCSCs with adult cardiomyocytes improves cardiomyocyte attachment and contractility. **A–B**. The number and percent of attached and beating cardiomyocytes from 7 -21 days following culture alone (Myo alone) or co-culture with either fibroblasts (Fibro+Myo), GATA-4 low expressing c-kit^pos^ cCSCs (cCSC1A+Myo, cCSC3C+Myo) or GATA-4 high c-kit^pos^ cCSCs (cCSC4A+Myo). *P<0.05 vs. Myo alone, Fibro+Myo, cCSC1A+Myo, cCSC3C+Myo. Data are Mean ± SD for 3 wells/condition and analysed using ANOVA. **C**. Representative bright field microscopy image showing co-culture of adult rat cardiomyocytes with GATA-4 high expressing cCSC4A at 7 days. Bar = 50 µm. **D–E**. The number and percent of attached and beating cardiomyocytes from 7–21 days following culture alone (Myo alone) or co-culture with either fibroblasts (Fibro+Myo), GATA-4 low expressing cCSCs(cCSC1A+Myo, cCSC3C+Myo) or GATA-4 high cCSCs (cCSC4A+Myo), using cell culture inserts to separate contact between the cCSCs and cardiomyocytes. *P<0.05 vs. Myo alone, Fibro+Myo, cCSC1A+Myo, cCSC3C+Myo. Data are Mean ± SD for 3 wells/condition and analysed using ANOVA. F. Representative bright field microscopy image showing adult rat cardiomyocytes on the substrate when GATA-4 high expressing cCSCs were cultured on the insert at 7 days. Bar = 50 µm.

We previously described that the presence of CSC-BC in the culture of adult ventricular rat cardiomyocytes led to enhancement of cardiomyocyte survival, over a 3 day culture period [20]. Here we wanted to assess whether this effect was sustained long-term and affected by different levels of GATA-4 expression in the cCSC populations. Therefore, we quantified the number of apoptotic cardiomyocytes, attached cardiac Troponin I (cTnI) positive adult cardiomyocytes and beating adult cardiomyocytes at 3, 7, 14 and 21 days after co-culture with either fibroblasts, c-kit^pos^ GATA-4 low or c-kit^pos^ GATA-4 high cCSCs. Co-culture of cardiomyocytes with c-kit^pos^ GATA-4 high cCSCs for 3 and 7 days, attenuated cardiomyocyte apoptosis measured by TdT assay and activated caspase-3 expression, compared to cardiomyocytes cultured alone and cardiomyocytes co-cultured with fibroblasts or c-kit^pos^ GATA-4 low cCSCs (Fig. 2A–F; Fig. S2). Cardiomyocytes were cultured on the substrate and c-kit^pos^ cCSCs were cultured on an insert to separate contact between the two cell populations and to allow for isolation of a pure, enriched cardiomyocyte preparation. Cardiomyocytes that were cultured with c-kit^pos^ GATA-4 low cCSC3C had increased expression of transcripts specific to cell death (i.e. Bax, Caspase-3, Fas) [30], [32]–[37] after 7 days in co-culture, compared to cardiomyocytes cultured together with c-kit^pos^ GATA-4 high cCSCs (Fig. 2G). The ratio of Bax and Bcl-2 can be used as a measure of apoptosis [37]. The Bax/Bcl-2 ratio was 3.0 for cardiomyocytes co-cultured with c-kit^pos^ GATA-4 low cCSCs, and 0.7 for cardiomyocytes co-cultured with c-kit^pos^ GATA-4 high cCSCs for 7 days. The co-culture of c-kit^pos^ GATA-4 high or c-kit^pos^ GATA-4 low cCSCs with cardiomyocytes had no effect on cardiomyocyte proliferation, measured by BrdU incorporation assay and Ki67 expression, over 7–21 days (Fig. 2H–I). As shown in Figure 3A–C and the supplemental online video S1, the number of attached and beating cardiomyocytes were increased in the co-culture with c-kit^pos^ GATA-4 high cCSCs (cCSC4A), at all time points analysed, compared to cardiomyocytes alone and co-culture with fibroblasts and c-kit^pos^ GATA-4 low expressing cCSCs (cCSC1A and cCSC3C). Another batch of c-kit^pos^ GATA-4 high cCSCs (cCSC10A) produced similar results when co-cultured with adult rat cardiomyocytes (Fig. S2). In order to determine if cell-to-cell contact impacts cardiomyocyte survival, we used cell culture inserts to separate cCSCs and cardiomyocytes. The number of adult cardiomyocytes that remained attached and sustained contraction over long term culture when separated by inserts, was also greatly enhanced by co-culture with c-kit^pos^ high expressing GATA-4 cCSC4A, compared to cardiomyocytes alone, co-culture with fibroblasts and co-culture with c-kit^pos^ low GATA-4 expressing cCSC populations (Fig. 3D–F; supplemental online video S2).

### Co-Culture of c-kit^pos^ GATA-4 high cCSCs with adult ventricular cardiomyocytes leads to increased IGF-1 expression

**Figure 4:**
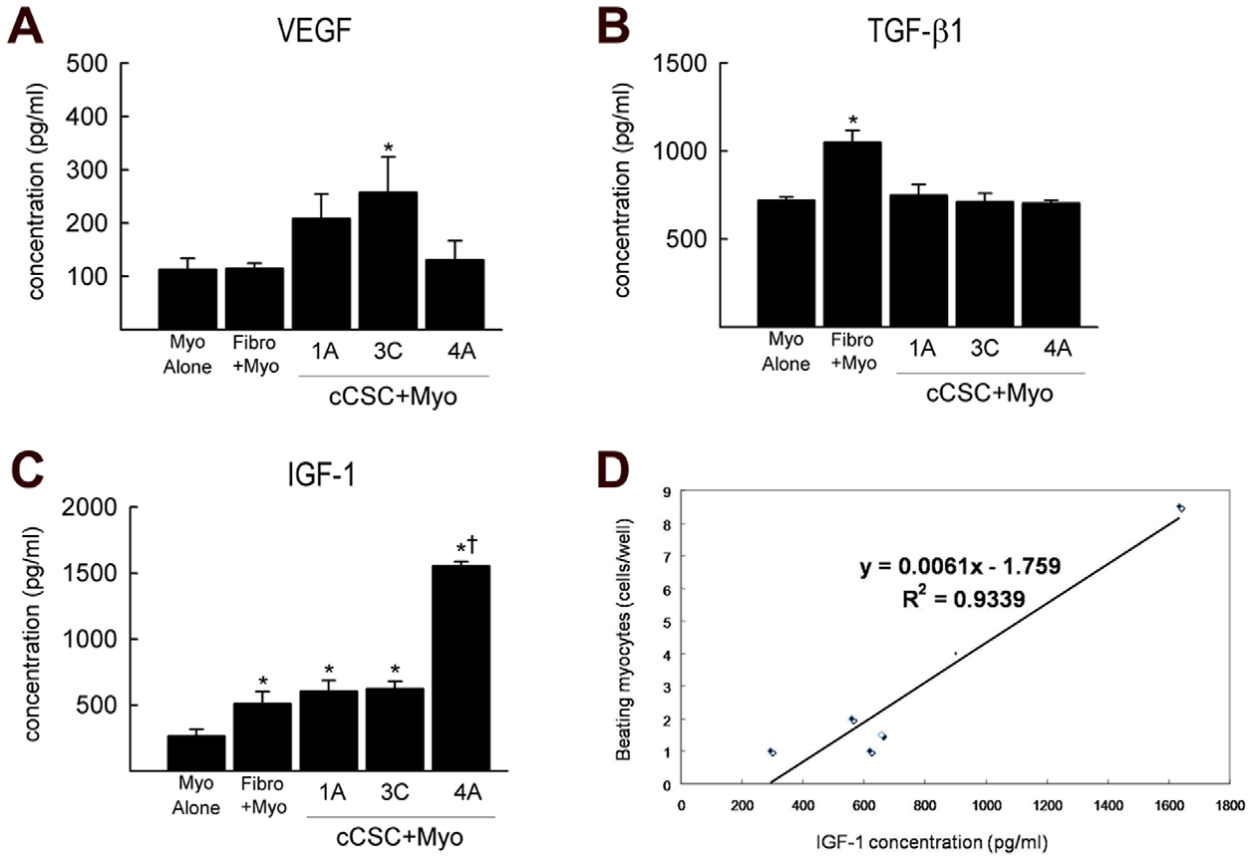
Increased IGF-1 concentration in the GATA-4 high expressing c-kit^pos^ cCSC/cardiomyocyte co-culture medium. **A**. VEGF concentration following 21 days of cardiomyocyte culture alone (Myo alone) or co-cultured with fibroblasts (Fibro+Myo), GATA-4 low expressing c-kit^pos^ cCSCs (cCSC1A+Myo, cCSC3C+Myo) or GATA-4 high c-kit^pos^ cCSCs (cCSC4A+Myo). *P<0.05 vs. Myo alone, Fibro+Myo, cCSC4A+Myo. Data are Mean ± SD of 3 assays (with 3 triplicates/assay) and analysed using ANOVA. **B**. TGF-β1 concentration following 21 days of cardiomyocyte culture alone (Myo alone) or co-cultured with fibroblasts (Fibro+Myo), GATA-4 low expressing c-kit^pos^ cCSCs (cCSC1A+Myo, cCSC3C+Myo) or GATA-4 high c-kit^pos^ cCSCs (cCSC4A+Myo). *P<0.05 vs. all. Data are Mean ± SD of 3 assays (with 3 triplicates/assay) and analysed using ANOVA. **C**. IGF-1 concentration following 21 days of cardiomyocyte culture alone (Myo alone) or co-cultured with fibroblasts (Fibro+Myo), GATA-4 low expressing c-kit^pos^ cCSCs (cCSC1A+Myo, cCSC3C+Myo) or GATA-4 high c-kit^pos^ cCSCs (cCSC4A+Myo). *P<0.05 vs. Myo alone, †P<0.05 vs. Fibro+Myo, cCSC1A+Myo, cCSC3C+Myo. Data are Mean ± SD of 3 assays (with 3 triplicates/assay) and analysed using ANOVA. D. IGF-1 concentration correlated with cardiomyocyte contractility. Correlation was determined using Pearson Product Moment Correlation coefficient.

In an attempt to ascertain whether certain survival growth factors are involved in cardiomyocyte survival when they were co-cultured with the different c-kit^pos^ GATA-4 expressing populations of cCSCs, we performed ELISA assay on the cCSC/cardiomyocyte co-culture medium for the cytokines IGF-1, VEGF, TGF-β1, TNF-α, and BMP-2. We found BMP-2 and TNF-α at very low levels (data not shown). VEGF was significantly up-regulated in the cCSC/cardiomyocyte co-culture medium when cardiomyocytes were co-cultured with c-kit^pos^ GATA-4 low expressing cCSC3C (Fig. 4A). TGF-β1 was increased in all conditions of cCSC/cardiomyocyte co-culture, but was significantly elevated when fibroblasts were co-cultured with cardiomyocytes (Fig. 4B). However, these increases did not correlate with cardiomyocyte survival, since these cCSC/cardiomyocyte co-cultures did not lead to longevity of cardiomyocyte survival or contraction (Figs. 2 and 3). We have shown a role for the TGF-β family in CSC functional differentiation when administered in a stage-specific protocol *in vitro* (Ellison et al. Unpublished data).

IGF-1 expression was significantly increased in the co-culture medium when cardiomyocytes were co-cultured with rat fibroblasts and both high- and low-expressing GATA-4 c-kit^pos^ cCSCs, compared to when cardiomyocytes were cultured alone (Fig. 4C). Interestingly, adult cardiomyocytes co-cultured with high GATA-4 expressing c-kit^pos^ cCSC4A lead to a significant 2 orders of magnitude increase of IGF-1 in the culture medium, compared to cardiomyocytes alone, co-culture with rat fibroblasts, and co-culture with c-kit^pos^ GATA-4 low expressing cCSC1A or cCSC3C (Fig. 4C). There was a strong correlation (r = 0.93) between the number of beating cardiomyocytes and IGF-1 expression level in the cCSC/cardiomyocyte co-culture medium (Fig. 4D).

**Figure 5:**
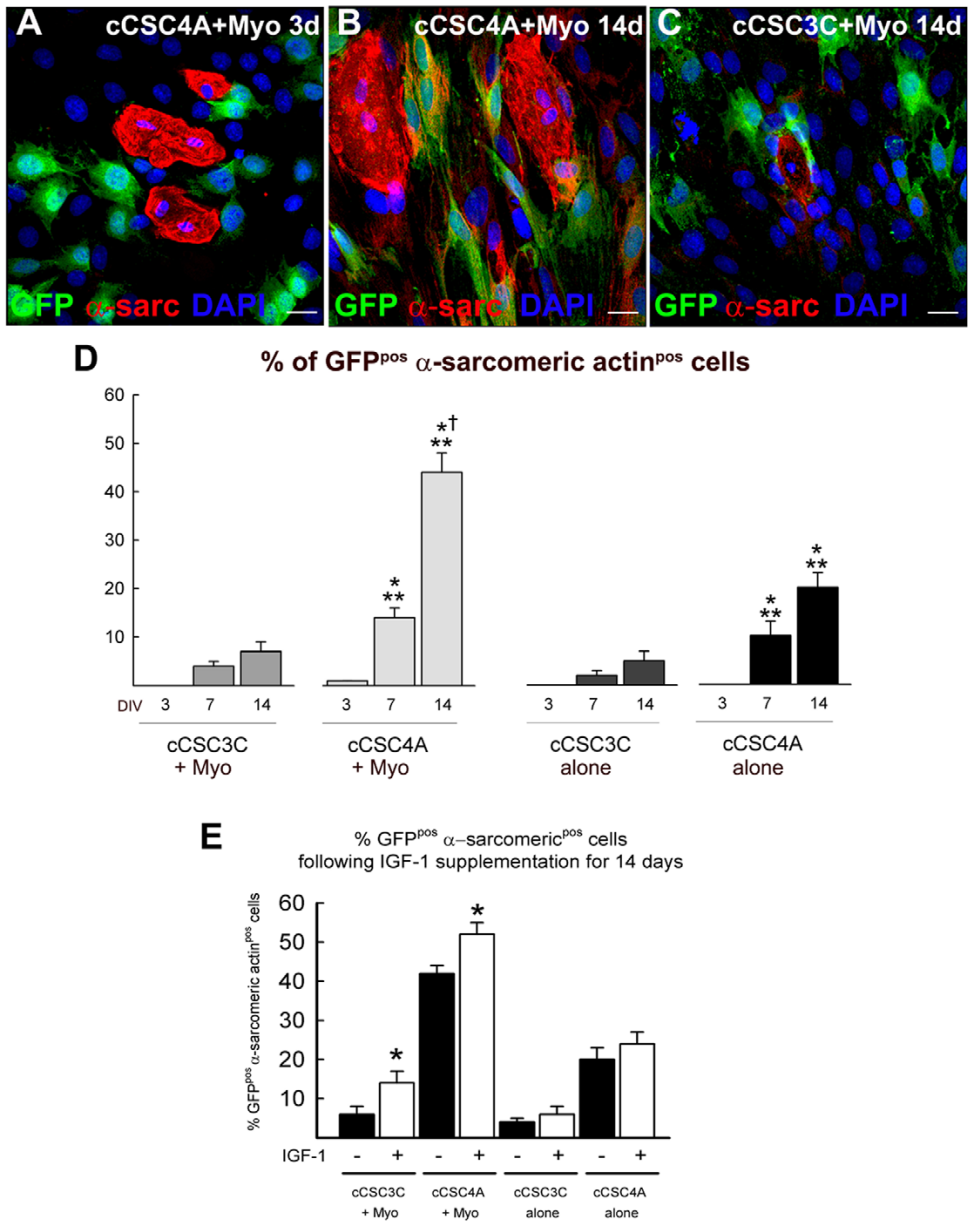
c-kit^pos^ GATA-4 high and low cCSC cardiomyogenic differentiation. **A–C**. Representative confocal microscopy images showing GFP^pos^ (green) GATA-4 high c-kit^pos^ cCSCs (cCSC4A+Myo) differentiated into the cardiomyocyte lineage (α-sarcomeric actin^pos^; red) when co-cultured with adult rat cardiomyocytes (α-sarcomeric actin^pos^; red) over 14 days (B). Note the lack of cardiomyogenic differentiation of GFP^pos^ (green) GATA-4 high c-kit^pos^ cCSCs at 3 days (cCSC4A+Myo; A) and GFP^pos^ (green) GATA-4 low c-kit^pos^ cCSCs at 14 days (cCSC3C+Myo; C). Bar = 20 µm. **D**. The percent number of GFP^pos^ α-sarcomeric actin^pos^ cells at 3, 7 and 14 days in vitro (DIV) following co-culture of GFP^pos^ GATA-4 low expressing c-kit^pos^ cCSCs with cardiomyocytes (cCSC3C+Myo), GFP^pos^ GATA-4 high c-kit^pos^ cCSCs with cardiomyocytes (cCSC4A+Myo) or culture alone (cCSC3C alone; cCSC4A alone). *P<0.05 vs. cCSC3C+Myo. ** vs. cCSC3C alone. † vs. cCSC4A alone. Data are Mean ± SD for 3 wells/condition and analysed using ANOVA. **E**. The percent number of GFP^pos^ α-sarcomeric actin^pos^ cCSC-derived cells following supplementation with IGF-1 for 14 days in either co-culture conditions (cCSC3C+Myo; cCSC4A+Myo) or culture alone (cCSC3C alone; cCSC4A alone). *P<0.05 vs. without (-) IGF-1. Data are Mean ± SD for 3 wells/condition and analysed using *t* test.

**Figure 6:**
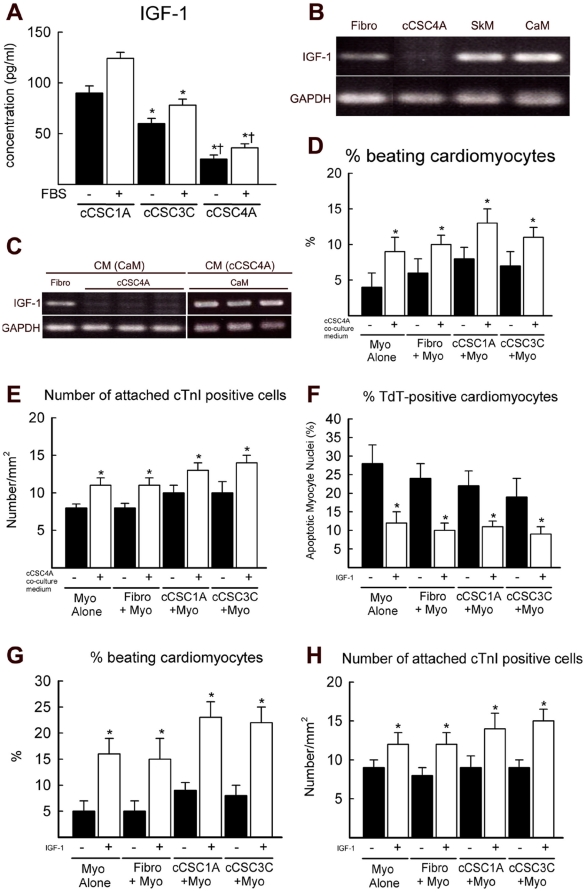
IGF-1 high concentration medium improves cardiomyocyte survival. **A**. ELISA assay assessed IGF-1 concentration in the culture medium, with and without FBS, of c-kit^pos^ GATA-4 low cCSC1A, cCSC3C and c-kit^pos^ GATA-4 high cCSC4A, when cultured alone. *P<0.05 vs. cCSC1A, †P<0.05 vs. cCSC3C. Data are Mean ± SD of 3 assays (with 3 triplicates/assay) and analysed using ANOVA. **B**. RT-PCR analysis showed c-kit^pos^ GATA-4 high cCSC4A had low IGF-1 message levels, compared to rat fibroblasts (Fibro), skeletal muscle cells (SkM) and cardiomyocytes (CaM). **C**. RT-PCR analysis showed that IGF-1 gene expression level in fibroblasts (Fibro) and c-kit^pos^ GATA-4 high cCSC4A (cCSC4A) is not altered through 7 days of culture in conditioned medium from adult cardiomyocytes (CM (CaM)). Also, IGF-1 gene expression level in cultured cardiomyocytes (CaM) is not altered through culture for 7 days with c-kit^pos^ GATA-4 high cCSC4A conditioned medium (CM (cCSC4A)). **D–E**. c-kit^pos^ GATA-4 high cCSC4A/cardiomyocyte co-culture conditioned medium, which is high in IGF-1 concentration, increased the percent number of beating cardiomyocytes and number of attached cTnI positive cells when cardiomyocytes were cultured alone (Myo Alone), or co-cultured with either fibroblasts (Fibro+Myo), or GATA-4 low expressing c-kit^pos^ cCSCs (cCSC1A+Myo, cCSC3C+Myo) for 7 days. *P<0.05 vs. without (-) cCSC4A co-culture conditioned medium. Data are Mean ± SD for 3 wells/condition and analysed using ANOVA. **F–H**. Supplementation of IGF-1 to the culture medium attenuated percent number of TdT-positive cardiomyocytes, increased percent number of beating cardiomyocytes and number of attached cTnI positive cells when cardiomyocytes were cultured alone (Myo Alone), or co-cultured with either fibroblasts (Fibro+Myo), or GATA-4 low expressing c-kit^pos^ cCSCs (cCSC1A+Myo, cCSC3C+Myo) for 7 days. *P<0.05 vs. without (−) IGF-1. Data are Mean ± SD for 3 wells/condition and analysed using ANOVA.

To ascertain if the co-culture conditions promoted cCSC cardiomyogenic differentiation, c-kit^pos^ GATA-4 high and low cCSCs were transfected with a lenti-virus encoding Green Fluorescent Protein (GFP) and co-cultured with cardiomyocytes for 14 days. c-kit^pos^ GATA-4 high cCSCs showed increased cardiomyogenic differentiation, when co-cultured with adult rat cardiomyocytes and when cultured alone, compared to c-kit^pos^ GATA-4 low cCSCs co-cultured with cardiomyocytes or when c-kit^pos^ GATA-4 low cCSCs were cultured alone (Fig. 5). However, the differentiated GFP-positive (GFP^pos^)/α-sarcomeric actin-positive (α-sarcomeric actin^pos^) cells were clearly morphologically distinguishable from the GFP-negative adult rat cardiomyocytes in the co-culture, at 3 through to 14 days (Figure 5A–C). Furthermore, GFP^pos^/α-sarcomeric actin^pos^ cells did not display rhythmic beating throughout the culture period. To determine if the increased IGF-1 in the c-kit^pos^ GATA-4 high cCSC/cardiomyocyte co-culture medium promoted commitment of cCSCs to the cardiomyocyte lineage, co-cultures of cardiomyocytes/c-kit^pos^ GATA-4 high cCSCs and cardiomyocytes/c-kit^pos^ GATA-low cCSCs were supplemented with 200 ng/ml recombinant IGF-1 for 14 days. Cultures of c-kit^pos^ GATA-4 high and c-kit^pos^ GATA-4 low cCSCs alone were also supplemented with 200 ng/ml IGF-1 for 14 days. The percentage of GFP^pos^/α-sarcomeric actin^pos^ cells was significantly increased when c-kit^pos^ GATA-4 high and c-kit^pos^ GATA-4 low cCSCs were co-cultured with cardiomyocytes supplemented with IGF-1, compared to when they were co-cultured without IGF-1 supplementation (Fig. 5E). IGF-1 supplementation had no effect on increasing cardiomyocyte differentiation of c-kit^pos^ GATA-4 high or c-kit^pos^ GATA-4 low cCSCs when cultured alone for 14 days (Fig. 5E). We then compared IGF-1 expression levels in the culture medium, when cCSCs were cultured alone. When assessed by ELISA assay and even when FBS was present in the culture medium, both c-kit^pos^ GATA-4 low and high expressing cCSCs released a small amount of IGF-1 (Fig. 6A), compared to cCSC/cardiomyocyte co-culture conditions (Fig. 4C). However, cCSC1A released more IGF-1, compared to cCSC3C and cCSC4A, and cCSC4A secreted significantly less compared to cCSC3C (Fig. 6A). Consistent with the ELISA results, IGF-1 at the message level in cCSC4A when cultured alone was extremely low, when compared to cardiomyocytes, skeletal muscle and rat fibroblasts (Fig. 6B). When cardiomyocytes were cultured in conditioned medium of c-kit^pos^ GATA-4 high cCSC4A there was no effect on IGF-1 gene expression level (Fig. 6C) and the conditioned medium from adult cardiomyocytes did not enhance the expression level of IGF-1 in c-kit^pos^ GATA-4 high cCSC4A or rat fibroblasts (Fig. 6C).

The conditioned medium of cardiomyocytes and c-kit^pos^ GATA-4 high cCSC4A co-culture, which is high in IGF concentration, did increase the percent number of beating cardiomyocytes when it was used on cardiomyocytes cultured alone (9±2%), cardiomyocytes co-cultured with fibroblasts (10±2%) and cardiomyocytes co-cultured with c-kit^pos^ GATA-4 low cCSCs (13±2 cCSC1A; 11±2 cCSC3C) for 7 days (Fig. 6D). Similar results were found for number of attached cTnI positive cardiomyocytes cultured alone, co-cultured with fibroblasts or c-kit^pos^ GATA-4 low cCSCs in conditioned medium of cardiomyocytes/c-kit^pos^ GATA-4 high cCSC4A (Fig. 6E). However, overall the effect on cardiomyocyte survival was not as great as when the cardiomyocytes were co-cultured with c-kit^pos^ GATA-4 high cCSC4A for 7 days (27±6% beating cardiomyocytes; 17±0.6 attached cTnI positive cardiomyocytes Fig. 3). When the conditioned co-culture medium from c-kit^pos^ GATA-4 low cCSCs was used on cardiomyocytes cultured alone, co-cultured with fibroblasts or c-kit^pos^ GATA-4 low cCSCs there was no effect on improving cardiomyocyte survival or contractility (Fig. S3). The increased expression of IGF-1 in the cCSC/cardiomyocyte co-culture medium with c-kit^pos^ GATA-4 high cCSC4A suggests that this population of cCSCs increases cardiomyocyte survival and contractility through an unknown mechanism, possibly due to increased GATA-4, which in turn regulates IGF-1 release. Indeed, other factors could also function as a mediator between cCSCs and cardiomyocytes.

Supplementation of 200 ng/ml recombinant IGF-1 to cardiomyocytes alone and co-cultures of cardiomyocytes/fibroblasts and cardiomyocytes/c-kit^pos^ GATA-4 low cCSCs significantly attenuated cardiomyocyte apoptosis, improved cardiomyocyte survival and contractility, measured by TdT assay, Caspase-3 expression, number of attached adult cardiomyocytes and percentage beating adult cardiomyocytes (Fig. 6E–H).

### IGF-1/IGF-1R/Akt pathway modulates the paracrine survival effect of cCSCs on adult cardiomyocytes

**Figure 7:**
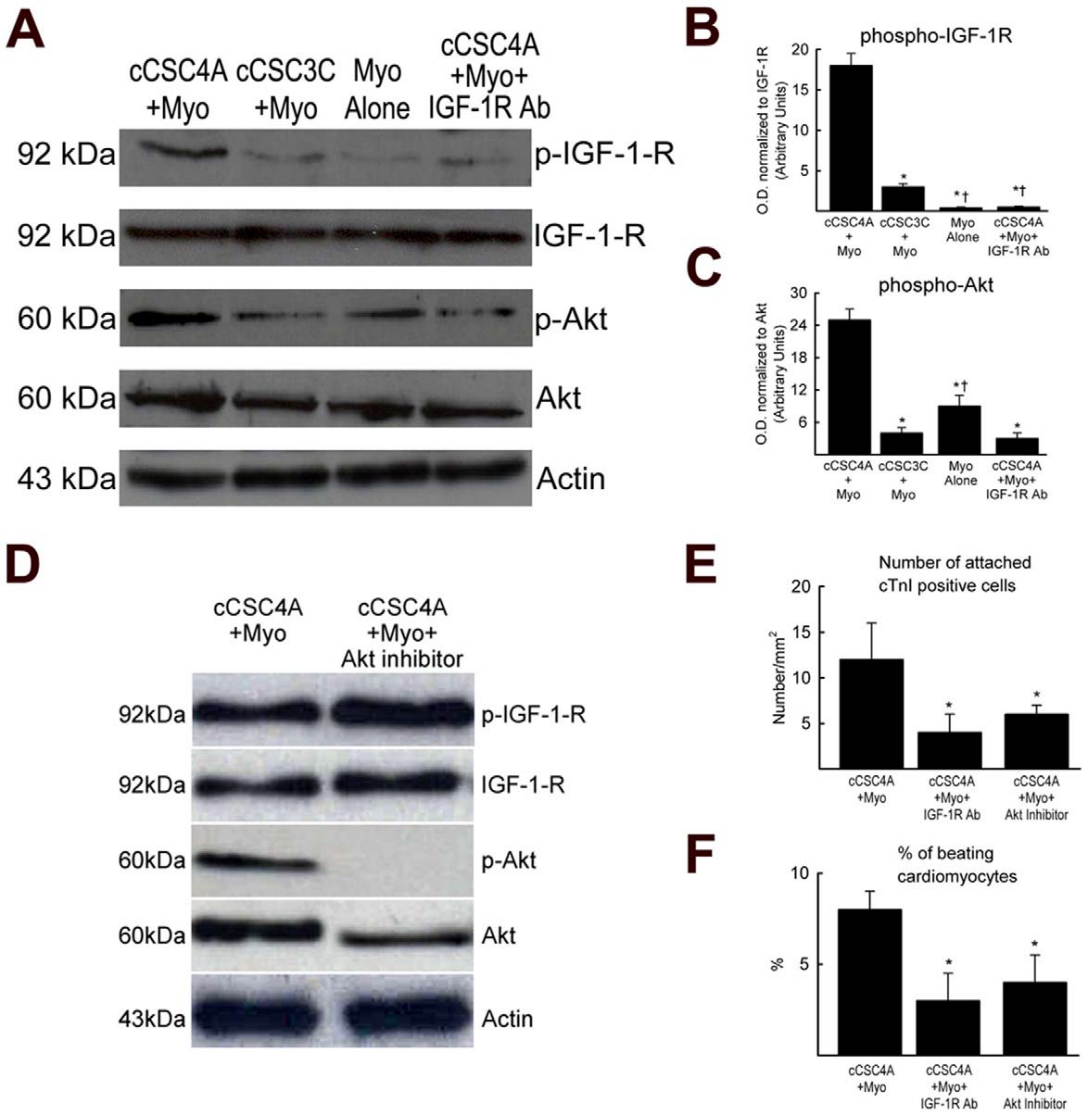
IGF-1 signalling explains the paracrine survival effect of GATA-4 high c-kit^pos^ cCSCs on adult cardiomyocytes. **A.** Representative Western blots show activation of the IGF-1 receptor (IGF-1-R) and downstream signaling to its physiological target, Akt, in cardiomyocytes co-cultured using inserts with c-kit^pos^ GATA-4 high cCSC4A (cCSC4A+Myo), but not in cardiomyocytes cultured alone (Myo Alone) or when co-cultured with c-kit^pos^ GATA-4 low cCSCs (cCSC3C+Myo) for 21 days. Addition of an IGF-1 receptor blocking antibody to the co-culture of c-kit^pos^ GATA-4 high cCSC4A plus cardiomyocytes obliterates IGF-1 signalling in the co-cultured cardiomyocytes. **B–C**. Optical Density (O.D.) of phospho-IGF-1R (B) and phospho-Akt (C). *P<0.05 vs. cCSC4A+Myo. †P<0.05 vs. cCSC3C+Myo in B. †P<0.05 vs. cCSC3C+Myo, cCSC4A+Myo+IGF-1R Ab in C. Data are Mean ± SD of 3 assays and analysed using ANOVA. **D**. Representative Western blots shows prevention of Akt phosphorylation in cardiomyocytes when the Akt inhibitor, 124005 was added to the cardiomyocyte/c-kit^pos^ GATA-4 high cCSC4A co-culture. **E–F**. Number of attached and percent number of beating cardiomyocytes decreased following inhibition of the IGF-1 signaling pathway through treatment with IGF-1 receptor blocking antibody or the Akt inhibitor, at day 19 for 48 hours. Data are Mean ± SD for 3 wells/condition and analysed using ANOVA. *P<0.05 vs. cCSC4A+Myo.

In order to ascertain that the increased IGF-1 in the co-culture medium of c-kit^pos^ GATA-4 high expressing cCSCs and adult cardiomyocytes is directly associated with improved cardiomyocyte survival, we harvested the cardiomyocytes from the substrate at 3 weeks after co-culture and analysed activation of the IGF-1 signaling pathway by Western blot. We found that the increased IGF-1 expression induced IGF-1 signaling as demonstrated by the increased IGF-1R and Akt phosphorylation in cardiomyocytes co-cultured with c-kit^pos^ GATA-4 high expressing cCSC4A, compared to cardiomyocytes alone and cardiomyocytes co-cultured with c-kit^pos^ GATA-4 low expressing cCSC3C (Figure 7A-D). Inhibition of the IGF-1 signaling pathway through treatment with IGF-1 receptor blocking antibody significantly inhibited cardiomyocyte survival in the c-kit^pos^ GATA-4 high expressing cCSC4A co-culture (Figure 7E–F) and, as expected, failed to induce IGF-1 signaling in cardiomyocytes co-cultured with c-kit^pos^ GATA-4 high expressing cCSC4A (Figure 7A–C). To expand on the role of IGF-1 signalling pathway on cardiomyocyte survival, treatment of cardiomyocyte/c-kit^pos^ GATA-4 high cCSC4A co-culture with the Akt inhibitor, 124005 prevented Akt phosphorylation (Fig. 7D) resulting in decreased cardiomyocyte survival and contractility (Fig. 7E–F). Together these data show that IGF-1/IGF-1R/Akt pathway modulates the paracrine survival effect of c-kit^pos^ GATA-4 high cCSCs on adult cardiomyocytes *in vitro*.

## Discussion

The main findings that emanate from the present study are: i) c-kit^pos^ cCSCs have differential expression of GATA-4. ii) The c-kit^pos^ cCSCs with high expression of GATA-4 enhance survival and contractility of adult cardiomyocytes when co-cultured *in vitro*. iii) The co-culture of high expressing GATA-4 c-kit^pos^ cCSCs with adult cardiomyocytes leads to increased IGF-1 level in the medium. iv) Increased IGF-1 expression correlates with increased cardiomyocyte survival and contractility in the cCSC/cardiomyocyte co-culture medium. v) IGF-1, and induction of its signalling pathway, modulates the paracrine survival effect of c-kit^pos^ GATA-4 high cCSCs on adult cardiomyocytes *in vitro*.

Cardiac stem cells (CSCs) are present in the adult mammalian heart at different physiological states [6], in that the CSC pool is composed of primitive cells expressing the pluripotent markers and also progenitor cells that have already committed to a specific lineage. We were able to generate clones of primitive or committed c-kit^pos^ CSCs, which had either low or high expression for GATA-4; the effects of these on adult cardiomyocyte survival *in vitro* were highly disparate. c-kit^pos^ GATA-4 high cCSCs enhanced the survival and contractility of adult rat ventricular cardiomyocytes when placed in a co-culture system over 3 weeks. This also resulted in an increase in the amount of IGF-1 in the cCSC/cardiomyocyte co-culture medium. These effects were not evident when adult rat cardiomyocytes were cultured without c-kit^pos^ cCSCs or when cultured with rat fibroblasts. Furthermore, other c-kit^pos^ cCSC populations, which expressed low levels of GATA-4, had no effect on cardiomyocyte survival and contractility or increased IGF-1 expression in the co-culture medium, suggesting a specific character of GATA-4 high expressing c-kit^pos^ cCSCs. These results fall short of conclusively proving a cause-effect relationship between GATA-4 expression, IGF-1 secretion and the protective effects of the c-kit^pos^ cCSCs on adult cardiomyocytes. However, the correlation is very strong since the phenotypes breed through in the subclones of both high and low GATA-4 expressors. Yet, to establish a firm causative relationship it will require knock-down and enhanced expression of GATA-4 on derivatives of the same line. Therefore, further investigation is warranted.

This increase of IGF-1 is considered to be from the result of co-culture with c-kit^pos^ GATA-4 high expressing cCSCs, because the ELISA assay and RT-PCR analysis showed low IGF-1 when c-kit^pos^ GATA-4 high cCSCs (cCSC4A) were cultured alone (Fig. 6). We observed no change in IGF-1 mRNA levels in cardiomyocytes after treatment with the conditioned medium from cCSC4A cells. However, the culture of adult cardiomyocytes with cCSC/cardiomyocyte co-culture c-kit^pos^ GATA-4 high cCSC4A conditioned media increased cardiomyocyte survival and contractility (Fig. 6D–E), but this was not as great when cardiomyocytes were co-cultured with c-kit^pos^ GATA-4 high expressing CSCs (Fig. 3). These results document that it is the condition of co-culture which stimulates the increased release of IGF-1 and resultant improved cardiomyocyte survival and contractility, and c-kit^pos^ GATA-4 high expressing cCSCs are more potent at delivering this effect. It seems there is a unique ‘cross-talk’, possibly related to increased GATA-4 expression, between the c-kit^pos^ GATA-4 high expressing cCSCs and the adult cardiomyocytes, fostering the increase and secretion of IGF-1 which in turn promotes cardiomyocyte survival and contractility. Furthermore, we have found a cross-talk between cardiomyocytes and CSCs and a growth factor para/autocrine loop fosters CSC growth and differentiation (Ellison et al. Unpublished data).

Supplementing IGF-1 to the culture medium of cardiomyocytes cultured alone and cardiomyocytes co-cultured with fibroblasts or c-kit^pos^ GATA-4 low cCSCs, where originally the level of IGF-1 is low, improved cardiomyocyte survival *in vitro* (Fig. 6F–H). IGF-1 was reported to promote cardiomyocyte survival *in vitro* via the Akt pathway [38]. Furthermore, IGF-1 over-expression in mice increased CSC and cardiomyocyte survival [21]. We show that the IGF-1 signaling pathway is activated and signaling to its downstream physiological targets in cardiomyocytes co-cultured with c-kit^pos^ GATA-4 high expressing cCSCs, compared to cardiomyocytes cultured alone and co-cultured with c-kit^pos^ GATA-4 low expressing cCSCs (Fig. 7A–D). When the IGF-1 pathway is switched off, due to blockade of the IGF-1 receptor with a specific antibody, cardiomyocyte survival is no longer apparent in the c-kit^pos^ GATA-4 high expressing cCSC/cardiomyocyte co-culture condition (Fig. 7E–F). Furthermore, when Akt phosphorylation is inhibited, survival of cardiomyocytes is decreased when co-cultured with c-kit^pos^ GATA-4 high cCSCs (Fig. 7D–F). These data document that IGF-1, and its signaling pathway, is a responsible paracrine factor in governing cardiomyocyte survival when co-cultured with c-kit^pos^ GATA-4 high expressing cCSCs. Our results also show that IGF-1 has pro-contractile properties, as the cardiomyocytes co-cultured with c-kit^pos^ GATA-4 high expressing cCSCs exhibited sustained rhythmic beating for up to 21 days and this was not evident in cardiomyocytes cultured alone or co-cultured with fibroblasts or c-kit^pos^ GATA-4 low expressing cCSCs (Fig. 2). Furthermore, sustained cardiomyocyte contraction persisted whether contact between the cells and cardiomyocytes was present or not. Previous findings have shown that IGF-1 over-expression and nuclear over-expression Akt transgenic mice have enhanced cardiomyocyte contractility and performance, due to increased cardiomyocyte shortening and velocity of shortening and re-lengthening coupled with a more efficient re-uptake of calcium by the sarcoplasmic reticulum (SR) [21], [39].

We and other groups have previously showed that IGF-1 and VEGF were specific growth factors that were elevated in the culture media and could therefore be associated with increased cardiomyocyte survival in co-culture conditions [20], [40]. Lai et al. (2009) demonstrated that BMDC possess potent myocardial protective properties and IGF-1R is required for this protection [41]. However, IGF-1 and IGF-2 supplementation did not affect creatine kinase release and cell death caused by ischemia/reoxygenation of human myocardial slices [41]. These findings would suggest that other factors are acting through the IGF-1R to produce the protective effects or that the role of IGF-1 and IGF-2 is necessary but not sufficient to achieve the benefit by BMDC and requires the concomitant effect of additional factor(s) [41]. In the present findings, it is also possible that as well as IGF-1, other factor(s) produced through the cCSC/cardiomyocyte co-culture conditions work through IGF-1/IGF-1R/Akt signaling pathway to improve cardiomyocyte survival.

The present findings show increased cardiomyocyte differentiation of c-kit^pos^ GATA-4 high cCSC4A when cultured alone in differentiation medium for 14 days (Fig. 5D). However, the differentiation is not complete as the cells do not exhibit sarcomeric structure or rhythmic beating. These data are consistent with our previous findings where c-kit^pos^ GATA-4 high cCSCs have an enhanced potential to differentiate into the cardiomyocyte lineage, compared to c-kit^pos^ GATA-4 low cCSCs [20]. IGF-1 showed no direct effect *per se* in promoting cardiomyocyte differentiation of c-kit^pos^ cCSCs when either GATA-4 high or low cCSCs were cultured alone (Fig. 5E). IGF-1 was up-regulated in the medium of cardiomyocytes co-cultured with c-kit^pos^ GATA-4 high cCSCs (Fig. 4C) and there was increased cardiomyocyte differentiation of the c-kit^pos^ GATA-4 high cCSCs at 7 and 14 days in this co-culture condition (Fig. 5D). Furthermore, when IGF-1 was supplemented to the co-culture of cardiomyocytes with c-kit^pos^ GATA-4 low and c-kit^pos^ GATA-4 high cCSCs there was increased cCSC differentiation into the cardiomyocyte lineage (Fig. 5E). Primarily, we showed that IGF-1 increased survival of cardiomyocytes in the co-culture with c-kit^pos^ GATA-4 high cCSCs (Figs. 2, 3, 4), and then also when IGF-1 was supplemented to the culture of cardiomyocytes alone, co-culture with fibroblasts and co-culture of cardiomyocytes with c-kit^pos^ GATA-4 low cCSCs (Fig. 6F–H). Taken together these data suggest increased cardiomyocyte survival is related to promoting differentiation of c-kit^pos^ cCSCs into the cardiomyocyte lineage *in vitro* (i.e. secretion of other factors by the cardiomyocytes). Recently, Field and colleagues [42] reported that c-kit^pos^ cells derived from adult mouse hearts fail to acquire a cardiomyogenic phenotype when co-cultured with fetal cardiomyocytes, and therefore questioning their regenerative potential. These data are at odds with several published reports [20], [43]–[46], including the results presented here. The data reported here were obtained with clonal c-kit^pos^ CSCs that are enriched for cardiomyogenic potential as c-kit^pos^ GATA-4 high cCSCs have significantly increased cardiomyogenic differentiation capacity compared to c-kit^pos^ GATA-4 low CSCs [20]. Furthermore, we have used adult derived cardiomyocytes and co-cultured them for up to 14 days, whereas Zaruba et al. (2010) used fetal cardiomyocytes and only co-cultured them with c-kit^pos^ cardiac cells for 7 days [42]. However, we report increased expression of sarcomeric protein in c-kit^pos^ GATA-4 high cCSCs co-cultured with adult rat cardiomyocytes yet we failed to detect sarcomeric structures, z-disc pattern or gap junction formations over 14 days (Fig. 5A–C), and therefore cCSC-derived functional beating cardiomyocytes in our CSC/cardiomyocyte co-culture system. Indeed, similar to cardiomyogenic differentiation of embryonic stem cells [47], we have identified an effective protocol utilizing key growth factors and cytokines which regulate c-kit^pos^ cCSC cardiomyogenic differentiation resulting in rhythmic contraction when administered in a stage-specific manner to cCSCs *in vitro* (Ellison et al. Unpublished).

Recent studies have focused on paracrine effects in adult stem cell therapy [48] and it is now the mechanism of choice to explain beneficial effects of BMDC cardiac cellular therapy. Indeed identification of the factors and molecules necessary for improving cardiomyocyte survival is of great relevance for cardiac regeneration studies. In the new era of regenerative medicine it is essential that we ascertain the ‘optimal’ type of cell to be used for regenerative myocardial therapies and a cell that has regenerative and renewal capacity, as well as exerting pro-survival and paracrine effects would be the ideal cell of choice. We previously reported and have also shown here that c-kit^pos^ GATA-4 high expressing cCSCs have an enhanced potential to differentiate into the cardiomyocyte lineage with potent cardiac regenerative capacity [20]. Taken together with the present findings, the c-kit^pos^ GATA-4 high CSCs could be the optimal cells for regenerative stem cell myocardial therapies. The ability of CSCs to instruct myocardial cell fate and function, by sustaining cardiomyocyte survival together with their regenerative action, could be of paramount importance to establish widely available allogeneic cell therapy for physiological and clinically meaningful myocardial regeneration and repair protocols [49].

In conclusion, clonal c-kit^pos^ CSCs, which can be multiplied in number at a fast rate, which express high levels of GATA-4 have a pro-survival effect on cardiomyocytes due to up-regulation of IGF-1 and resultant IGF-1 signalling pathway activation when co-cultured with adult cardiomyocytes. These effects were not apparent when c-kit^pos^ GATA-4 low expressing clonal CSCs were used. Therefore, these findings extend the knowledge of CSCs as having a paracrine role yet identify for the first time the specific CSC population which is responsible for having this pro-survival effect.

## Supporting Information

Table S1 qPCR primers
(0.04 MB DOC)

Figure S1 Representative live cell image showing adult cardiomyocytes cultured on the substrate (A) and GFP^pos^ (green) c-kit^pos^ cCSCs cultured on the insert (B). There is no contamination of GFP^pos^ c-kit^pos^ cCSCs on the substrate in A. Bar = 50 µm.
(0.55 MB JPG)

Figure S2 Co-culture of cardiomyocytes with another batch c-kit^pos^ GATA-4 high cCSCs (cCSC10A) also attenuated cardiomyocyte apoptosis measured by TdT assay (A) and activated caspase-3 (B) expression, and improved cardiomyocyte attachment (C) and contractility (D).
(1.01 MB JPG)

Figure S3 c-kit^pos^ GATA-4 low cCSC3C/cardiomyocyte co-culture conditioned medium, which is low in IGF-1 concentration did not improve the number of attached cTnI positive cells (A) or percent of beating cardiomyocytes (B) when cardiomyocytes were cultured alone (Myo Alone), or co-cultured with either fibroblasts (Fibro), or GATA-4 low expressing c-kit^pos^ cCSCs clones (cCSC1A, cCSC3C) for 7 days.
(0.28 MB JPG)

Video S1 Representative video showing beating cardiomyocytes co-cultured with GATA-4 high cCSCs.
(1.86 MB AVI)

Video S2 Representative video showing beating cardiomyocytes co-cultured with GATA-4 high cCSCs, separated by culture inserts.
(1.97 MB AVI)
