# Supplementary material for: c-kitpos GATA-4 High Rat Cardiac Stem Cells Foster Adult Cardiomyocyte Survival through IGF-1 Paracrine Signalling
Source: PLoS One. 2010 Dec 13;5(12):e14297. doi: 10.1371/journal.pone.0014297 (PMC3001457; doi:10.1371/journal.pone.0014297)
Supplement: Table S1 — qPCR primers (0.04 MB DOC) [file pone.0014297.s001.doc]

| *Primer Name* | ***Sequence*** | ***Product (bp)*** | ***Accession*** |
| --- | --- | --- | --- |
| GATA-4 forward  GATA-4 reverse | CTGTGCCAACTGCCAGACTA  AGATTCTTGGGCTTCCGTTT | 165 | [NM_144730](http://www.ncbi.nlm.nih.gov/entrez/viewer.fcgi?db=nucleotide&val=25282464) |
| IGF-1 forward  IGF-1 reverse | cagttcgtgtgtggaccaag  tcagcggagcacagtacatc | 151 | [BC086374](http://www.ncbi.nlm.nih.gov/nuccore/BC086374) |
| BCL2 forward  BCL2 reverse | CGACTTTGCAGAGATGTCCA  ATGCCGGTTCAGGTACTCAG | 223 | [NM_016993](http://www.ncbi.nlm.nih.gov/nucleotide/8392973) |
| BAX forward  BAX reverse | TGCAGAGGATGATTGCTGAC  GATCAGCTCGGGCACTTTAG | 173 | [NM_017059.1](http://www.ncbi.nlm.nih.gov/nucleotide/8392964) |
| FASL forward  FASL reverse | TGCCTCCACTAAGCCCTCTA  AGGCTGTGGTTGGTGAACTC | 166 | [NM_012908.1](http://www.ncbi.nlm.nih.gov/nuccore/6978524?from=74&to=910&report=gbwithparts) |
| Caspase3 forward  Caspase3 reverse | GGACCTGTGGACCTGAAAAA  GCATGCCATATCATCGTCAG | 159 | [NM_012922.2](http://www.ncbi.nlm.nih.gov/nucleotide/52138591) |
| PARP1 forward  PARP1 reverse | AGTAAAGAAGCTGGCGGTGA  ATAGAGTAGGCGGCCTGGAT | 183 | [NM_013063.2](http://www.ncbi.nlm.nih.gov/nucleotide/55742841) |
| GAPDH forward  GAPDH reverse | CTCATGACCACAGTCCATGC  TTCAGCTCTGGGATGACCTT | 155 | [NM_017008.3](http://www.ncbi.nlm.nih.gov/nucleotide/110347607) |
| ACTIN forward  ACTIN reverse | AGCCATGTACGTAGCCATCC  CTCTCAGCTGTGGTGGTGAA | 228 | [NM_031144.2](http://www.ncbi.nlm.nih.gov/nucleotide/42475962) |

**Table S1.** qPCR primers
