# Supplementary figures and images for: c-kitpos GATA-4 High Rat Cardiac Stem Cells Foster Adult Cardiomyocyte Survival through IGF-1 Paracrine Signalling
Source: PLoS One. 2010 Dec 13;5(12):e14297. doi: 10.1371/journal.pone.0014297 (PMC3001457; doi:10.1371/journal.pone.0014297)

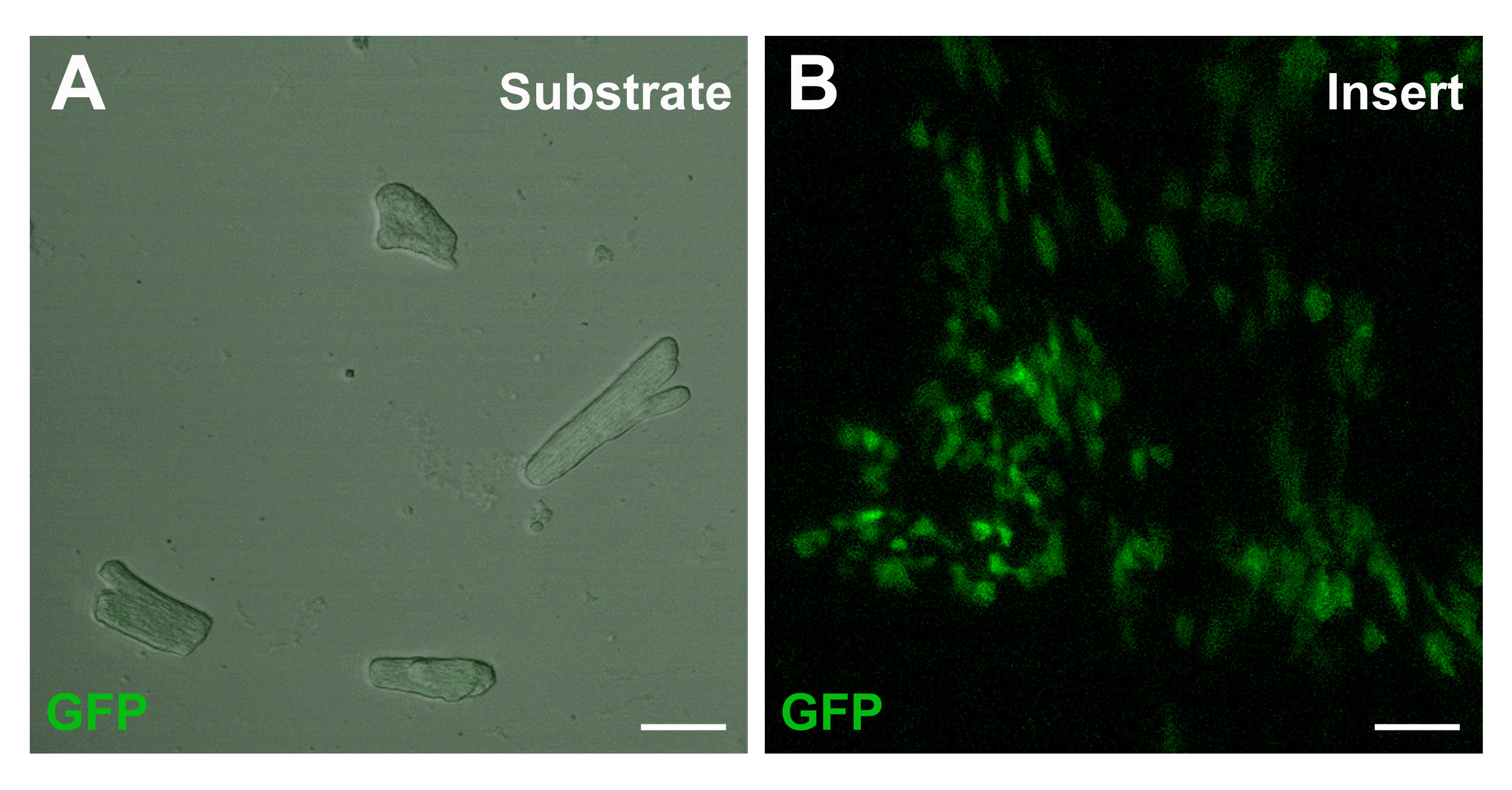

Supplement: Figure S1 — Representative live cell image showing adult cardiomyocytes cultured on the substrate (A) and GFPpos (green) c-kitpos cCSCs cultured on the insert (B). There is no contamination of GFPpos c-kitpos cCSCs on the substrate in A. Bar = 50 µm. (0.55 MB JPG) [file pone.0014297.s002.jpg]

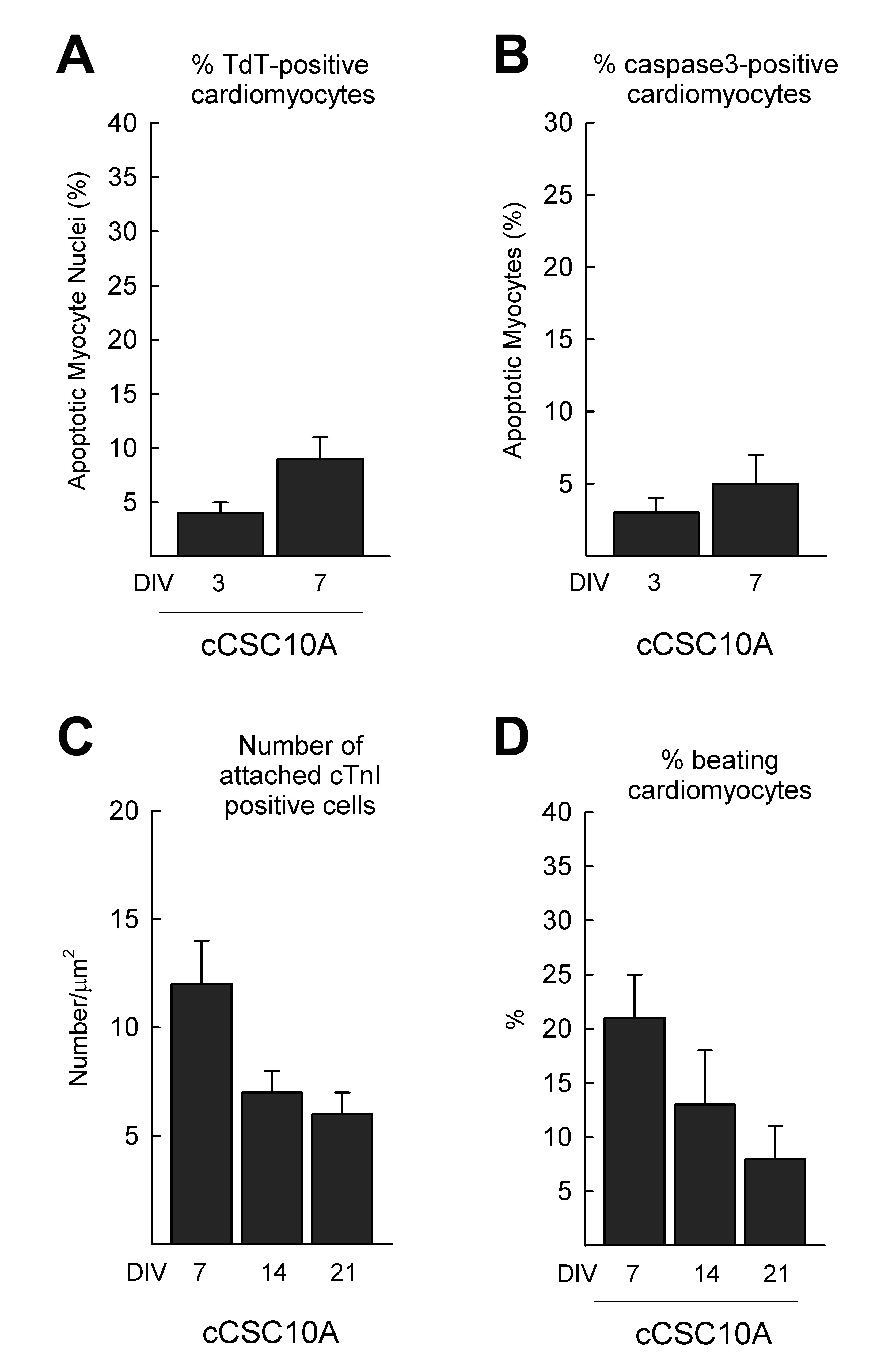

Supplement: Figure S2 — Co-culture of cardiomyocytes with another batch c-kitpos GATA-4 high cCSCs (cCSC10A) also attenuated cardiomyocyte apoptosis measured by TdT assay (A) and activated caspase-3 (B) expression, and improved cardiomyocyte attachment (C) and contractility (D). (1.01 MB JPG) [file pone.0014297.s003.jpg]

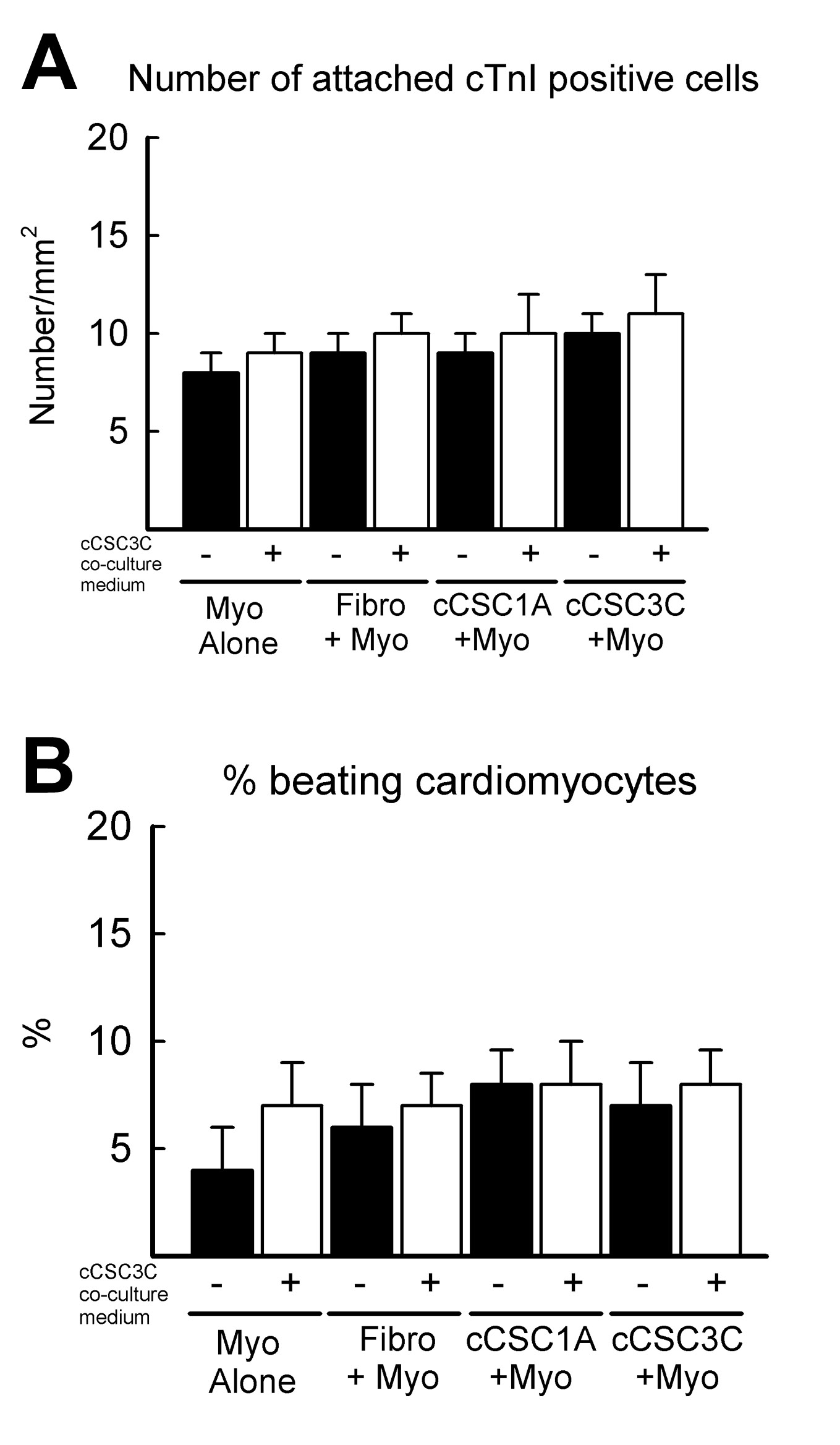

Supplement: Figure S3 — c-kitpos GATA-4 low cCSC3C/cardiomyocyte co-culture conditioned medium, which is low in IGF-1 concentration did not improve the number of attached cTnI positive cells (A) or percent of beating cardiomyocytes (B) when cardiomyocytes were cultured alone (Myo Alone), or co-cultured with either fibroblasts (Fibro), or GATA-4 low expressing c-kitpos cCSCs clones (cCSC1A, cCSC3C) for 7 days. (0.28 MB JPG) [file pone.0014297.s004.jpg]
